# Topological transitions of the generalized Pancharatnam-Berry phase

**DOI:** 10.1126/sciadv.adg6810

**Published:** 2023-11-24

**Authors:** Manuel F. Ferrer-Garcia, Kyrylo Snizhko, Alessio D’Errico, Alessandro Romito, Yuval Gefen, Ebrahim Karimi

**Affiliations:** ^1^ Nexus for Quantum Technologies, University of Ottawa, Ottawa, K1N 5N6 ON, Canada.; ^2^ Department of Condensed Matter Physics, Weizmann Institute of Science, Rehovot 76100, Israel.; ^3^ Institute for Quantum Materials and Technologies, Karlsruhe Institute of Technology, 76021 Karlsruhe, Germany.; ^4^ Université Grenoble Alpes, CEA, Grenoble INP, IRIG, PHELIQS, 38000 Grenoble, France.; ^5^ Department of Physics, Lancaster University, Lancaster LA1 4YB, UK.

## Abstract

Distinct from the dynamical phase, in a cyclic evolution, a system’s state may acquire an additional component, a.k.a. geometric phase. Recently, it has been demonstrated that geometric phases can be induced by a sequence of generalized measurements implemented on a single qubit. Furthermore, it has been predicted that these geometric phases may exhibit a topological transition as a function of the measurement strength. We demonstrate and study this transition experimentally by using an optical platform where the qubit is represented by the polarization of light and the weak measurement is performed by means of coupling with the spatial degree of freedom. Our protocol can be interpreted in terms of environment-induced geometric phases, whose values are topologically determined by the environment-system coupling strength. Our results show that the two limits of geometric phase induced by sequences of either weak or projective measurements are topologically distinct.

## Teaser

Geometric phases are experimentally shown to exhibit different topologies depending on the measurement strength.

## INTRODUCTION

When a quantum state undergoes a cyclic evolution, the phase acquired is given by the well-known dynamical component and an additional contribution, associated with the geometrical features of the path followed by the state. This additional contribution is known as the geometric phase. The general framework for the emergence of a geometric phase has been pointed out first by Berry (*1*) in the context of adiabatic quantum evolution. A specific realization of this phase had been earlier considered by Pancharatnam (*2*) in his study of generalized interference theory. Pancharantnam’s theory shows how geometric phases can be acquired in a nonadiabatic cyclic evolution, noting that these are given by the area enclosed by the respective trajectory of the system in the state space. The Pancharantnam phase can be observed following a sequence of running projective measurements, each of a different observable, where the last measurement projects on the initial state (*3*–*5*). Geometric phases have found applications in several fields of physics (*6*), in particular, in optics (*7*–*10*) and condensed matter physics (*11*–*15*). The Berry phase is a key theme for understanding topological phases of matter (*15*). For instance, the Berry phase plays the role of a topological invariant in one-dimensional chiral symmetric systems (*16*, *17*) and serves as the fundamental building block in the definition of other topological invariants, such as Chern numbers (*18*). Going beyond Hamiltonian dynamics, the emergence of geometric phases has been predicted and observed in the context of non-Hermitian evolution (*19*–*21*); these phases were further shown to emerge following a sequence of weak measurements (*22*, *23*). Further pursuing the latter theme, a major theoretical development has revealed that dynamics comprising multiple measurements may assign topological features to geometric phases. In particular, the limits of weak and strong measurement are topologically distinct (*23*–*25*). This prediction has recently been confirmed using a superconducting qubit platform (*26*). That study has implemented postselection on each individual measurement. This aligns with the original theoretical proposal (*23*–*25*), yet it leaves the question open: To what extent is the predicted topological transition a feature of the specific laid-down protocol?

The experiment reported here not only uses a platform different from that of (*26*) (namely, an optical platform) but also introduces a conceptually different protocol: rather than exercising postselection on each individual detector’s readout, here, we implement postselection on a joint readout of all measurements of the run. We find that a topological phase transition also takes place under such generalized conditions, with distinct values of the topological number characterizing the respective limits of projective and infinitely weak measurements. Our experimental procedure consists of a sequence of measurements, each implemented by a set of optical elements. The key optical element is a polarization-sensitive beam displacer (BD), which is used to execute a weak measurement of the polarization state of a laser beam. Using additional elements [quarter-wave plates (QWPs) and compensating wave plates (CWPs)] serves to tune the measurement to a specific observable. The strength of the measurement is determined by the ratio of the beam width and the difference of transverse displacements of orthogonal polarizations. The detector’s readout is, in fact, the polarization degree of freedom of the photon, which, in turn, could be viewed as the system, while the transverse position can be viewed as the environment. Our protocol could then be interpreted as an environment inducing a geometric phase, highlighting the dual nature of detector/environment. Last, we investigate the robustness of the observed topological properties with respect to setup imperfections.

## RESULTS

### Theoretical overview

**Fig. 1.**
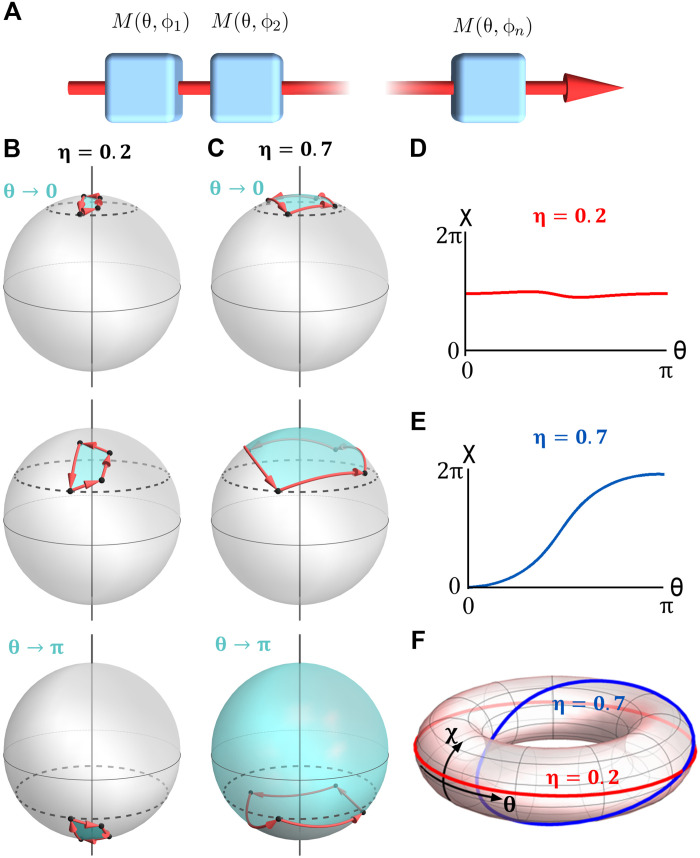
Measurement-induced phase and its topological transition. (**A**) The state trajectory of a system is determined by a series of *N* measurements along different directions (θ, ϕ). (**B** and **C**) The state trajectory on the Bloch sphere for a sequence of three measurements with strength η = 0.2 (B) and η = 0.7. (C) Different rows represent different values of θ. The black dashed line corresponds to θ, at which the measurements are performed. The black points connected by red arrows denote the system state trajectory, as induced by the measurements. The colored portion of the Bloch sphere is the solid angle subtended by the respective trajectory. The accumulated geometric phase on each trajectory is plotted in (**D** and **E**) as a function of θ. At both points θ = 0 and π, χ mod 2π = 0, making these values of θ equivalent. Hence, the curves can be mapped onto a torus (**F**), which highlights the topological distinction between the cases η = 0.2 and η = 0.7.

We consider a class of processes where *N* measurements are performed on a quantum system, as shown in Fig. 1A. Each step is a postselected measurement associated with the polarization state ∣θ, ϕ⟩, where θ and ϕ stand for the polar and azimuthal coordinates on the Bloch sphere, respectively. We can define a sequence of measurements (θ, ϕ*_n_*) for a fixed value of θ ∈ [0, π] while the azimuth is spanned in discrete steps ϕ_*n*_ = 2π*n*/(*N* + 1). Let us denote the acquired geometric phase χ_η_(θ), where η ∈ [0, ∞) is introduced to indicate the strength of the measurement. It can be shown that Δχ_η_ = χ_η_(π) − χ_η_(0) = 2π*m*, where *m* is an integer; see Supplementary Materials for more details.

As illustrated in Fig. 1B, for infinitely weak measurements, η → 0, the effect of each measurement is vanishingly small. Therefore, χ(θ) = 0 for any value of θ, implying that Δχ_η→0_ = 0. However, in the limit of projective measurements, strong measurement, one observes that Δχ_η→∞_ = 2π, as shown in Pancharatnam’s geometric-phase theory (*2*). An example of the latter case, strong measurement limit, is illustrated in Fig. 1C: When θ = 0, the measurement sequence does not change the projected state. Thus, the trajectory on the Bloch sphere shrinks to a single point independently of the measurement strength. In consequence, the enclosed area—and the geometrical phase—is zero. For θ → 0, the state follows a loop close to the initial projected state, acquiring a small geometric phase. As θ → π, the state follows a similar loop close to the south pole of the Bloch sphere, thus the enclosed geometric phase is close to 2π. This gives Δχ_η→∞_ = 2π, as stated above. The distinction between Δχ_η→0_ = 0 and Δχ_η→∞_ = 2π suggests the existence of a transition in the behavior of the geometric phase, as the measurement strength η is varied. Because Δχ = 2π*m*, the nature of the transition is topological.

The topological nature of this transition becomes evident when plotting the function χ ∈ [0,2π) as a function of θ ∈ [0, π] (Fig. 1, D to F). Because χ(0) = χ(π) = 0(mod 2π), this function can be mapped onto a closed loop on the torus *T* = [0,2π) × [0, π), as shown in Fig. 1F. For Δχ = 2π (sufficiently large η), one obtains a curve that wraps once around the vertical cycle of the torus, while for Δχ = 0 (sufficiently small η), the corresponding curve can be continuously deformed in the coordinate curve χ = 0. The two curves obtained in the strong and weak measurement cases cannot be continuously deformed into each other. Therefore, the dependence of the geometric phase on θ in the strong and weak measurement regimes is topologically distinct.

Note that the topological transition is not possible if the function χ(θ) is always well defined and continuous. At the critical measurement strength, η_cr_, the function χ_cr_(θ) is not well defined. The studies in (*23*–*25*) predict that this happens via a vanishing interference contrast at some θ at η_cr_. Below, we confirm this in our experiments.

### Experimental setup

**Fig. 2.**
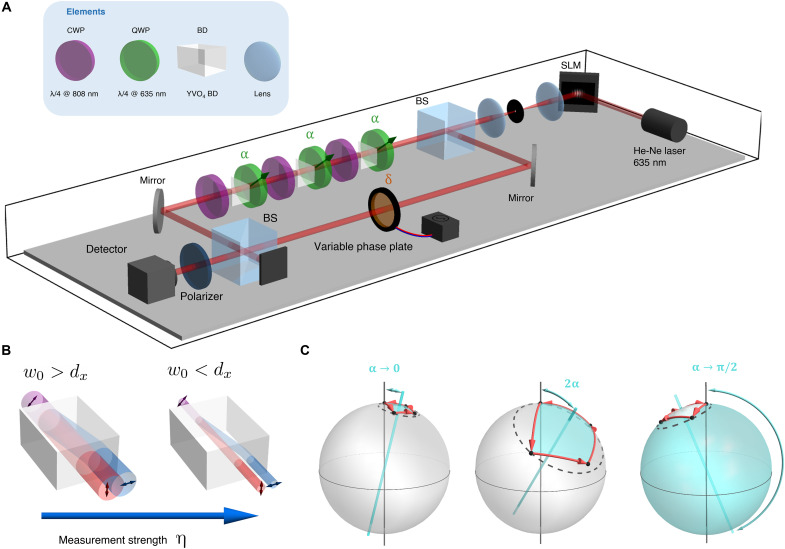
Optical implementation of a sequence of weak polarization measurements. (**A**) Experimental setup used to detect the geometrical phase acquired because of a sequence of polarization measurements. A 632.9-nm laser emits a vertically polarized Gaussian beam that impinges on a spatial light modulator (SLM) to obtain a beam with a certain width *w*_0_. The beam is split into two paths: in one is subjected to a sequence of transformations, while a spatially uniform phase δ is applied to the other path. Each stage is composed of a QWP, whose fast axis is oriented at angle α with respect to the vertical, a BD, and a QWP for 808 nm acting as the compensation wave plate (CWP). Lastly, the output power of the interference is recorded after the recombined beam passes through a vertical polarizer. BS, beam splitter. (**B**) The measurement strength η = *d_x_*/*w*_0_ is controlled by varying the waist parameter *w*_0_ of the input beam. When *w*_0_ is much larger than the beam displacement *d_x_*, the displacement is ineffectual, corresponding to a weak measurement. For *w*_0_ < *d_x_*, the two polarizations become two well-separated beams, leading to a projective measurement in the limit *w*_0_ → 0. (**C**) In contrast to the examples exhibited in Fig. 1 (B and C), the sequence of measurements produced by this setup corresponds to a circle of θ = 2α, which is additionally rotated by 2α around the *x* axis of the Bloch sphere. This does not affect the subtended area and, consequently, the accumulated geometric phase.

We demonstrate the existence of this topological transition in an optical experiment where the qubit state is associated with the polarization of a coherent beam. As illustrated in Fig. 2A, the beam goes through a series of *N* = 3 identical optical stages that emulate the measurement steps. Each stage is composed of a quarter wave plate (QWP), whose fast axis is oriented at angle α = θ/2 with respect to the vertical, followed by a YVO_4_ beam displacer (BD) and an additional compensation wave plate (CWP). The BD’s ordinary and extraordinary axes are aligned along $\hat{y}$ and $\hat{x}$, respectively. Therefore, the BD shifts the centroid of the horizontally polarized component by a distance *d_x_*, keeping the vertically polarized contribution unchanged. The BD essentially performs measurement on the vertical/horizontal polarization basis, as the horizontally polarized component of the beam is spatially displaced. If the beam waist *w*_0_ is larger than *d_x_*, then the measurement is weak, because there is no sharp separation between the two polarization components (see Fig. 2B). If the waist is much smaller than the displacement, *w*_0_ ≪ *d_x_*, then this implements a projective measurement, as the two polarization components are completely separated. Therefore, we can control the measurement strength by modifying *w*_0_. The CWP with a vertically aligned fast axis is used to compensate for the phase difference between the two polarization components accumulated while propagating inside the BD.

The role of the QWPs is to implement the desired sequence of measurement directions (θ, ϕ*_n_*) on the Bloch sphere. The rotation by angle α enables controlling the polar angle θ of the measurement axis. The sequence of measurements induced by the setup in Fig. 2A corresponds to the directions (θ, ϕ*_n_*) rotated by an angle θ = 2α around the *y* axis of the Bloch sphere; cf. Fig. 2C. The details of this correspondence are explained in the “Simplifying the experimental setup” section. Given the geometric nature of the induced phase, the expected topological transition remains unaffected by this rotation, both qualitatively and quantitatively. Lastly, to complete the cyclic evolution, the polarization state is projected onto the initial state using a polarizer. Our aim is to investigate the geometrical phase acquired by the undeflected beam (corresponding to the measurement postselected to yield a null outcome). This is done by interfering the final state with the reference beam, which only experiences a controllable phase shift δ introduced by a variable phase plate. The output power at the interferometer exit is recorded as a function of δ. The shift of this curve corresponds to the acquired geometric phase. The input beam is generated by means of a spatial light modulator that displays a hologram allowing to tailor the beam waist through the technique introduced in (*27*). We set the input beam’s polarization state to be vertical ($\hat{y}$), corresponding to the initial state in the direction (θ, ϕ_0_) in the theoretical protocol. On the basis of this setup, the measurement protocol to unveil the hidden topological transition is given as follows. The strength of our intermediate *N* = 3 measurements is regulated by varying the waist parameter of the input beam: The value of *w*_0_ is inversely proportional to the measurement strength η = *d_x_*/*w*_0_ (see Fig. 2C). For a fixed waist parameter, we proceed to get power readouts as a function of the reference arm phase shift δ ∈ [0, 2π], while α = θ/2 is kept constant. From here, it is possible to retrieve the accumulated geometrical phase χ_η_(θ = 2α) for a given orientation of QWPs, α, by proper curve fitting. By varying the QWP orientation, it is possible to reconstruct the behavior of χ_η_(θ = 2α) for all α for a given measurement strength.

### Experimental results

**Fig. 3.**
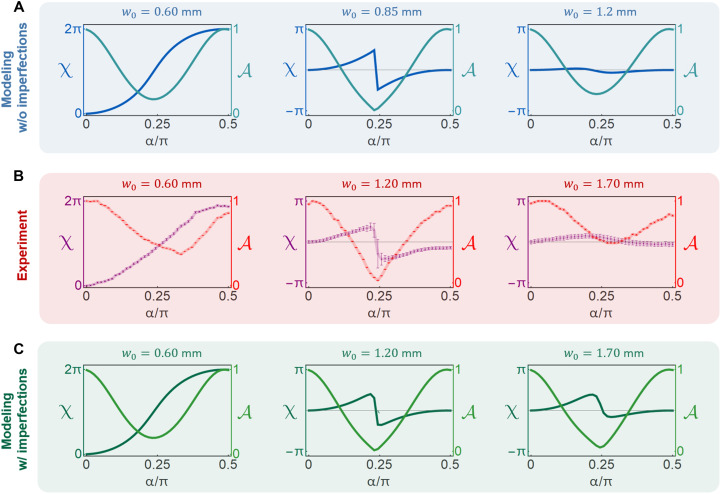
Experimentally measured and theoretically simulated geometric phase. Topological transition in the measurement-induced geometric phase χ(α = θ/2): (**A**) theoretical modeling, (**B**) experimental results, and (**C**) modeling incorporating the imperfection of the birefringent crystals. The plots show the phase χ(α) and the interference contrast A. The left column corresponds to a narrow beam (small *w*_0_, strong measurement) and features Δχ = 2π. The right column corresponds to a large beam width (weak measurement) and exhibits Δχ = 0. The middle column represents a point close to the transition: The phase χ(α) exhibits a sharp change near α = π/4. The sharp change of the phase coincides with the vanishing of the interference contrast, which renders χ(α) ill-defined and enables the topological transition.

Here, we discuss the experimental results and their relation to the theoretical predictions. Because the postselection in our experiment goes beyond the original theoretical proposal, we have modeled the experiment to confirm the presence of a topological transition theoretically (see the “Optical implementation of the null-weak measurement” section). Figure 3A shows that when *w*_0_ is sufficiently small, i.e., strong measurement regime, the simulation predicts Δχ = χ(α = π/2) − χ(α = 0) = 2π, while for the case of weak measurements (*w*_0_ < *d_x_*), Δχ = 0. A sharp transition occurs at *w*_0_ = 0.85 mm, where the interference contrast vanishes for α ≈ π/4, enabling the abrupt change of the phase behavior. The experiment was carried on by performing measurements for *w*_0_ between 0.6 and 2.5 mm. The experimental results, shown in Fig. 3B, clearly exhibit a similar transition between Δχ = 2π for small *w*_0_ and Δχ = 0 for large *w*_0_, as well as the vanishing contrast at the transition.

The difference Δχ = χ(π/2) − χ(0) in the observations is not strictly equal to 0 or 2π but can slightly deviate from these values. This is seen most prominently for *w*_0_ = 0.6 mm. We attribute this to the stability of the Mach-Zehnder interferometer, in particular to a small drift in the phase between the two arms during the measurement process (which was performed in 45 min). We emphasize that this does not violate the topological quantization of Δχ but introduces an error in its extraction. In all the cases, the extracted Δχ is close to either 0 or 2π, making the determination of the topological index *m* straightforward. The vanishing contrast at the transition also confirms the expected phenomenology of the topological transition.

We note that the waist $w_{0}^{*}$ at which the transition happens clearly deviates from the theory predictions: $w_{0}^{*}$ = 0.85 mm in the simulation, while $w_{0}^{*}$ = 1.2 mm in the experiment. We attribute this deviation to the fact that the surfaces of the BDs are parallel within a few tens of arc seconds, as stated by the manufacturer and verified by us independently. This tiny angle between the two surfaces induces a small transverse wave vector difference between the two components. We have incorporated this effect into our theoretical modeling, the results of which are presented in Fig. 3C. With this, we are able to reproduce the change in the transition location. A detailed analysis of these imperfections and the enhanced modeling can be found in section S2.

**Fig. 4.**
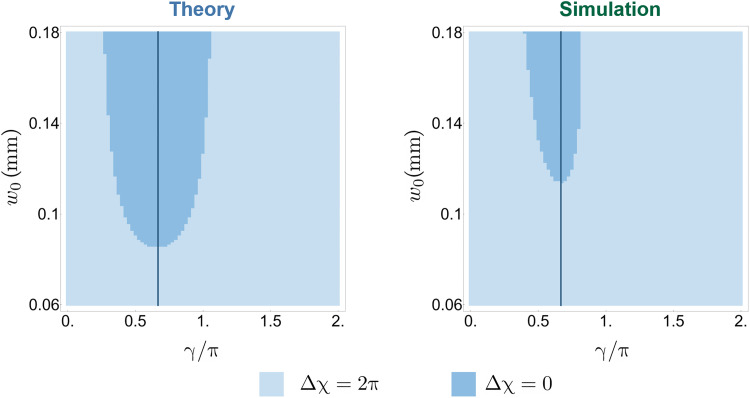
Topological phase dependence on compensating waveplates. The phase diagram (theoretical) depicting the topological properties of the measurement-induced phase as a function of beam waist *w*_0_ and the compensation phase γ. We present the results of a theory simulation without (left) and including (right) the experimental imperfections of the birefringent crystals. Note that the trivial phase with (Δχ = 0) exists only in a narrow interval of the phase compensation parameter The vertical line indicates the parameters used in our experiment. The imperfections of birefringent crystals clearly make the trivial region shrink yet do not eliminate the topological transition.

Theoretical studies have predicted (*24*, *25*) that the topological transition only exists if the dynamical phases are compensated accurately enough. In our work, this condition is satisfied. In Fig. 4 we explored theoretically the topological phase diagram considering the additional parameter γ corresponding to the optical retardation of the CWP. The results show that there is a range of values of γ where the topological transition can be observed, both in the ideal scenario and in the case of imperfect optical elements.

## DISCUSSION

We have demonstrated that measurement-induced geometric phases in optical systems exhibit a topological transition. In particular, we consider a family of processes parameterized by a variable α and a measurement strength η. We demonstrated that the geometrical phase, with respect to η and α, exhibits a nontrivial topology. More precisely, the variation Δχ in the geometrical phase as a function of α undergoes a sharp transition of 2π as η is varied. The parameter η can be viewed as the coupling strength with an environment, here represented by the light’s spatial degree of freedom. In this framework, our observations can be interpreted as topological transitions induced by the coupling to an external environment. The topological transition is robust to amending the protocol and the imperfections in the measurement process. The location of the topological transition depends on specific details of the system (quality of BDs, retardation of the compensating wave plates, etc.). This sensitivity of the transition location may be useful for characterizing optical elements or for sensing. We leave this, however, to future investigations.

## MATERIALS AND METHODS

### Optical implementation of the null-weak measurement

In our optical implementation of the measurements, the detectors are two-state systems with possible readouts *r* = ±. We use the photon spatial degree of freedom, i.e., its location in the *xy* plane (transverse to the propagation direction). The formal description of this is as follows. The incident photon’s electric field can be described as

$$E_{0}(x,y)=\left(\begin{array}{c} E_{0y}\\ E_{0x} \end{array}\right)\sqrt{\frac{2}{\pi w_{0}^{2}}}e^{-(x^{2}+y^{2})/w_{0}^{2}}e^{ikz}$$ (1)

The measurement is implemented via a BD (see Fig. 2B) that shifts the *x*-polarized component in space

$$E(x,y)\propto \left(\begin{array}{c} e^{ikn_{y}L_{y}}E_{0y}e^{-(x^{2}+y^{2})/w_{0}^{2}}\\ e^{ikn_{x}L_{x}}E_{0x}e^{-({\left[x-d_{x}\right]}^{2}+y^{2})/w_{0}^{2}} \end{array}\right)e^{ikz}$$ (2)

where normalizing factors are not shown. Apart from the displacement, the phases associated with propagation in the BD, *kn_x_L_x_* and *kn_y_L_y_*, are imprinted onto the polarization components. The overall phase is not important, whereas the difference γ = *kn_x_L_x_* − *kn_y_L_y_* may lead to observable consequences; cf. Fig. 4. In our protocol, we compensate for this phase difference; see below. Therefore, here we put, for simplicity, *kn_x_L_x_* = *kn_y_L_y_* = 0.

If, after experiencing the BD, the beam were to interfere with the original beam, then the interference term would be

$$\int dx\;dy\;E_{0}^{*}(x,y)E(x,y)={|E_{0y}|}^{2}+{|E_{0x}|}^{2}e^{-\frac{d^{2}}{2w_{0}^{2}}}$$ (3)

$$={\left(\begin{array}{c} E_{0y}\\ E_{0x} \end{array}\right)}^{†}M_{-}\left(\begin{array}{c} E_{0y}\\ E_{0x} \end{array}\right)$$ (4)

where *M*_−_, in analogy with the notation in the Supplementary Materials, is the diagonal matrix $\mathrm{diag}(1,\sqrt{1-\zeta })$, with $\sqrt{1-\zeta }=e^{-d_{x}^{2}/(2w_{0}^{2})}$. Therefore, a BD implements a postselected null weak measurement in the photon’s polarization space. The measurement strength $\eta =\sqrt{-\ln(1-\zeta )}=d_{x}/w_{0}$, as defined in the section "Experimental setup." The limit of projective measurement corresponds to η → ∞, while the infinitely weak measurement corresponds to η → 0.

Note that in our actual setup (cf. Fig. 2A), the interference happens after three beam displacements have been performed. Therefore, the postselection is implemented not on the readout of each individual measurement but on the combined “readout” of all measurements. This constitutes an important conceptual difference compared to the original definition of the measurement-induced phase and its topological transition, detailed in the Supplementary Materials. Observation of the topological transition in our work, thus, underlines that the transition is not a feature of a specific narrow protocol but a more general phenomenon.

#### Phase difference compensation

To compensate for the unwanted phase difference γ = *kn_x_L_x_* − *kn_y_L_y_*, one can use a phase plate

$$P(\varphi )=\left(\begin{array}{cc} e^{i\varphi /2} & 0\\ 0 & e^{-i\varphi /2} \end{array}\right)$$ (5)

Choosing ϕ = γ and placing the phase plate after the BD leads to

$$\begin{array}{c} P(\gamma )\;\mathrm{BD}\;E_{0}(x,y)=\sqrt{\frac{2}{\pi w_{0}^{2}}}e^{ik(n_{x}L_{x}+n_{y}L_{y})/2}e^{ikz}\\ \times \left(\begin{array}{c} E_{0y}e^{-(x^{2}+y^{2})/w_{0}^{2}}\\ E_{0x}e^{-({\left[x-d_{x}\right]}^{2}+y^{2})/w_{0}^{2}} \end{array}\right) \end{array}$$ (6)

leaving one only with an unimportant overall phase. The overall phase is unimportant because it does not depend on the incoming polarization and thus can be calibrated away.

In our setup (cf. Fig. 2A), the required phase compensation is implemented with a QWP for a wavelength distinct from that of the laser we use. We denote it as CWP.

#### Measuring different observables

The measurement procedure described above leads to the back action matrix *M*_−_, i.e., to measuring σ*_z_*. To implement measurements of different observables **n** · **σ**, corresponding to **n** = (sinθcosϕ, sinθsinϕ, cosθ), one needs to be able to (i) discriminate different linear polarizations (not only horizontal and vertical) with a BD and (ii) convert elliptical polarizations to linear and back, so that they can be discriminated by the BD.

(i) can be implemented by rotating the BD in the *xy* plane

BD(θ/2)=R(θ/2)BDR(−θ/2) (7)

with the rotation matrix

$$R(\theta /2)=\left(\begin{array}{cc} \cos\theta /2 & -\sin\theta /2\\ \sin\theta /2 & \cos\theta /2 \end{array}\right)$$ (8)

(ii) can be implemented by placing phase plates *P*(± ϕ) before and after the BD.

Therefore, a measurement of **n** · **σ** can be implemented via a sequence of elements that involves a rotated BD and CWP, as well as two phase plates

M(θ,ϕ)=P(−ϕ)R(θ/2)P(γ)BDR(−θ/2)P(ϕ) (9)

Note that to rotate the measurement axis by θ, one needs to perform real space rotations by α = θ/2.

This sequence uses four elements per measurement, whereas our setup in Fig. 2 features only three optical elements per measurement. We describe how this is achieved in the next section.

### Simplifying the experimental setup

The protocol for observing the topological transition requires sending in a laser beam with polarization

$$E_{in}=\left(\begin{array}{c} E_{0y}\\ E_{0x} \end{array}\right)=\left(\begin{array}{c} \cos\theta /2\\ \sin\theta /2 \end{array}\right)=R(\theta /2)\left(\begin{array}{c} 1\\ 0 \end{array}\right)$$ (10)

and using *N* measurements M(θ, ϕ*_j_*), where the measurement stages are defined in Eq. 9 and ϕ*_j_* = 2π*j*/(*N* + 1). The number of required optical elements can be reduced. To do this, one needs two observations.

First, consider the incoming polarization and the first measurement

$$\begin{array}{c} M(\theta ,\phi_{1})\left(\begin{array}{c} \cos\theta /2\\ \;\sin\theta /2 \end{array}\right)=P(-\phi_{1})R(\theta /2)P(\gamma )\;\mathrm{BD}\\ \times \underbrace{R(-\theta /2)P(\phi_{1})R(\theta /2)}\left(\begin{array}{c} 1\\ 0 \end{array}\right) \end{array}$$ (11)

The block *R*(−θ/2)P(ϕ_1_)*R*(θ/2) can be interpreted as a phase plate rotated by the angle α = θ/2, P(ϕ_1_, α).

Second, consider two sequential measurements

$$\begin{array}{c} M(\theta ,\phi_{j+1})M(\theta ,\phi_{j})=P(-\phi_{j+1})R(\theta /2)P(\gamma )\;\mathrm{BD}\\ \times \underbrace{R(-\theta /2)P(\phi_{j+1})P(-\phi_{j})R(\theta /2)}\\ \times P(\gamma )\;\mathrm{BD}\;R(-\theta /2)P(\phi_{j}) \end{array}$$ (12)

The block *R*(−θ/2)P(ϕ_*j*+1_)P(−ϕ*_j_*)*R*(θ/2) can be replaced with a single rotated phase plate P(ϕ_*j*+1_ − ϕ*_j_*, α) = P(2π/(*N* + 1), α) = P(ϕ_1_, α).

Therefore, instead of having a rotated incoming polarization and rotated BDs, one can have vertical incoming polarization and rotated phase plate P(ϕ_1_, α) before the BDs. Note that this setup simplification involves replacing all phase plates P(ϕ*_j_*) with their rotated versions *R*(−θ/2)P(ϕ*_j_*)*R*(θ/2) and the input polarization *R*(θ/2)(1 0)^T^ with ${\left(1\;0\right)}^{T}$. The simplified setup is related to the original protocol by rotating all the measurement axes **n***_j_* by angle θ around the *y* axis of the Bloch sphere. For our choice of *N* = 3, we have ϕ_1_ = π/2, making the required phase plates P(ϕ_1_, α) QWPs and leading to the setup in Fig. 2A.

## Supplementary Materials

### This PDF file includes:

Sections S1 and S2 Fig. S1 References
