# Supplementary material for: Topological transitions of the generalized Pancharatnam-Berry phase
Source: Sci Adv. 2023 Nov 24;9(47):eadg6810. doi: 10.1126/sciadv.adg6810 (PMC10672158; doi:10.1126/sciadv.adg6810)
Supplement: Supplementary file 1 — Sections S1 and S2 Fig. S1 References [file sciadv.adg6810_sm.pdf]

Science Advances
AAAS

Supplementary Materials for

Topological transitions of the generalized Pancharatnam-Berry phase

Manuel F. Ferrer-Garcia *et al.*

Corresponding author: Alessio D'Errico, aderrico@uottawa.ca

Sci. Adv. **9**, eadg6810 (2023)
DOI: 10.1126/sciadv.adg6810

This PDF file includes:

Sections S1 and S2
Fig. S1
References

Section 1. THEORETICAL PROTOCOL AND ITS RELATION TO THE EXPERIMENTAL SETUP

Here we provide a brief theoretical background on quantum measurement theory, on measurement induced geometric phases, and the topological transition in them, as well as connect the quantum measurement formalism to the optical setup employed in the paper.

A. Null-weak measurements of different observables in the polarisation space

The formalism applies to the transition reported in the main text when regarding the polarisation of the laser beam as a quantum polarisation state $|\theta,\phi\rangle = \cos(\theta/2)\left|\uparrow\right\rangle + e^{i\phi}\sin(\theta/2)\left|\downarrow\right\rangle$ of a photon, where $\left|\uparrow\right\rangle$ and $\left|\downarrow\right\rangle$ label the linearly independent vertical and horizontal polarisations respectively with $\theta \in [0,\pi]$ and $\phi \in [0, 2\pi)$.

In the most general setting, a measurement of a quantum system in a state $|\psi\rangle$ returns an outcome r with probability $P(r) = \langle\psi|M_r^\dagger M_r|\psi\rangle$, while the state is updated as $|\psi\rangle \to |\psi'\rangle = M_r\,|\psi\rangle/\sqrt{P(r)}$. The process is controlled by the Kraus operators M_r, which depend on the specific detection process and fulfil $\sum_r M_r^\dagger M_r = 1$ due to overall probability conservation [28]. For what we are concerned here, we specialize in a measurement process, known as null weak measurement, with two possible outcomes, $r = +, -$, and corresponding Kraus operators

$$M_+ = \sqrt{\zeta}\left|\downarrow\right\rangle\left\langle\downarrow\right|, \quad M_- = \left|\uparrow\right\rangle\left\langle\uparrow\right| + \sqrt{1-\zeta}\left|\downarrow\right\rangle\left\langle\downarrow\right|, \tag{S1}$$

where $0 \le \zeta \le 1$ corresponds to the measurement strength $\eta = \sqrt{-\ln(1-\zeta)}$. For $\zeta = 1$ ($\eta \to \infty$), the measurement is projective: the operation projects the state on $\left|\downarrow\right\rangle$ if $r = +$ and $\left|\uparrow\right\rangle$ if $r = -$. For $\zeta < 1$, however, we either have a collapse to $\left|\downarrow\right\rangle$ ("click", described by M_+) or no collapse ("no-click" or "null measurement", described by M_-). In the latter case, the system's state is updated to a the post-measurement state, which depends on the pre-measurement one.

The measurement procedure in Eq. (S1) corresponds to an imperfect, or weak, measurement of σ_z. This can be generalized to measure arbitrary observables, i.e. to distinguish two arbitrary basis states. A direction $\mathbf{n} = (\sin\theta\cos\phi, \sin\theta\sin\phi, \cos\theta)$ is identified by the polar and azimuthal angles θ and ϕ on the Bloch sphere. The corresponding orthogonal basis states, $\left|\uparrow_\mathbf{n}\right\rangle$ and $\left|\downarrow_\mathbf{n}\right\rangle$, are defined as eigenstates of $\mathbf{n}\cdot\boldsymbol{\sigma}$ associated with the respective eigenvalue ±1. For $\phi = 0$ and π these states correspond to two mutually orthogonal linear polarisations, whereas for all other values of ϕ they correspond to general elliptic polarisations. The analog of Eq. (S1) is then given by $M_+(\mathbf{n}) = \sqrt{\zeta}\left|\downarrow_\mathbf{n}\right\rangle\left\langle\downarrow_\mathbf{n}\right|$, $M_-(\mathbf{n}) = \left|\uparrow_\mathbf{n}\right\rangle\left\langle\uparrow_\mathbf{n}\right| + \sqrt{1-\zeta}\left|\downarrow_\mathbf{n}\right\rangle\left\langle\downarrow_\mathbf{n}\right|$.

B. Measurement-induced geometric phases

In order to induce a geometric phase, one needs to use multiple measurements. We label them by $j = 1, ..., N$. Each measurement is determined by the observable it measures, i.e., by the direction $\mathbf{n}_j$. The respective Kraus operators are $M_{r_j}^{(j)} = M_{r_j}(\mathbf{n}_j)$, where $r_j = \pm$ is the readout of measurement j.

For each such measurement with a given outcome, the phase of the post-measurement state is gauge-dependent, hence non-physical. However, if a sequence of measurements leads to a post-measurement final state $|\psi_f\rangle$ which is proportional to the initial one, $|\psi_0\rangle$, the phase difference between $|\psi_f\rangle$ and $|\psi_0\rangle$ is a legit observable given by

$$\chi_{\{r_j\}} = \arg\langle\psi_0|\psi_f\rangle = \arg\langle\psi_0|\, M_{r_N}^{(N)} \dots M_{r_2}^{(2)} M_{r_1}^{(1)}\,|\psi_0\rangle\,, \tag{S2}$$

where r_j is the outcome (readout) of the j-th measurement, whose effect is encoded in the Kraus operator $M_{r_j}^{(j)}$.

Note that even if the final state, $|\psi_f\rangle$, differs from the initial one, the phase defined in Eq. (S2) is still well-defined: this can be understood by introducing a fake projective measurement onto the initial state, $|\psi_0\rangle\langle\psi_0|$, after the application of all $M_{r_j}^{(j)}$, in order to force $|\psi_f\rangle \propto |\psi_0\rangle$. Therefore, Eq. (S2) defines a legitimate observable for a general sequence of measurements. The sequence of (normalized) post-measurement intermediate states, $|\psi_0\rangle$, $M_{r_1}^{(1)}\,|\psi_0\rangle$, $\dots$, $M_{r_N}^{(N)} \dots M_{r_1}^{(1)}\,|\psi_0\rangle$, defines a trajectory on the Bloch sphere. This trajectory is given by the set of geodesics connecting the states. For Hermitian Kraus operators, $M_{r_j}^{(j)\dagger} = M_{r_j}^{(j)}$ (which is the case in Eq. (S1)), the measurement-induced phase, (S2), has a geometric interpretation as $\chi_{\{r_j\}} = \Omega/2$, where Ω is the solid angle subtended by the trajectory on the Bloch sphere [22-25].

C. Protocol for topological transition in measurement-induced phases

Consider a family of measurement sequences, defined in the main text. Each sequence consists of N measurements corresponding to $(\theta, \phi_j = 2\pi j/(N+1))$, where $j = 1, ..., N$ is the measurement number. The family is obtained when considering such sequences at all $\theta \in [0, \pi]$. We perform postselection, which restricts measurement readouts to be $r_j = -$ for all j. According to Eq. (S2), this defines a phase $\chi_{\{-\}}(\theta)$ for each measurement sequence in the family. For briefness, we denote this phase $\chi(\theta)$.

The function $\chi(\theta)$ possesses a topological invariant. This follows from the fact that at $\theta = 0$ and $\theta = \pi$, $M_{r_j}^{(j)} |\psi_0\rangle = |\psi_0\rangle$ for all j, implying that $|\psi_f\rangle = |\psi_0\rangle$. That is, the measurements do not change the system state so that the resulting phase is trivial: $e^{i\chi(0)} = e^{i\chi(\pi)} = 1$. As with any phase, the measurement-induced phase is defined up to an integer multiple of 2π. Without loss of generality, we can set $\chi(0) = 0$. This, however, eliminates the freedom of adding multiples of 2π at all other θ due to the natural demand of continuity of $\chi(\theta)$. In particular, $\chi(\pi)$ may be non-zero; yet $e^{i\chi(\pi)} = 1$ implies that $\chi(\pi) = 2\pi m$ with integer m. In other words, the difference $\Delta\chi = \chi(\pi) - \chi(0)$, must be quantized in units of 2π, as stated in the main text. Further, the quantization of $\Delta\chi$ implies that its value cannot be changed by continuous deformations of the function $\chi(\theta)$. Therefore, $\Delta\chi$ constitutes a topological invariant.

As discussed in the main text, for infinitely weak ($\zeta \to 0$) and projective ($\zeta \to 1$) measurements $\Delta\chi_{\zeta\to 0} = 0$ and $\Delta\chi_{\zeta\to 1} = 2\pi$ respectively. This necessitates a jump in the topological invariant, i.e., a topological transition, at some critical measurement strength, $\zeta_c \in [0, 1]$.

D. Detection of measurement-induced geometric phases

In order to detect a measurement-induced phase, one needs to interfere the state that underwent measurements with the initial unmeasured state, as in Fig. 2a of the main text. Here we provide a theoretical description of this in the quantum measurement formalism.

The incoming photon in polarisation state $|\psi_0\rangle$ becomes, after the beam splitter, the state $|\psi\rangle = \frac{1}{\sqrt{2}} |\psi_0\rangle \otimes (|0\rangle + |1\rangle)$ where $|0\rangle$ and $|1\rangle$ refer to the two arms of the interferometer. A sequence of measurements with Kraus operators $M_{r_j}(\mathbf{n}_j)$ is performed in the interferometer arm denoted as $|0\rangle$. The arm denoted as $|1\rangle$ features only a phase shifter, δ. Therefore, after passing through the respective interferometer arms the photon state is

$$|\psi\rangle_{int} = \frac{1}{\sqrt{2}} \left[\sum_{\{r_j\}} |\Psi_0(\{r_j\})\rangle \otimes |0\rangle + |\Psi_1\rangle \otimes |1\rangle \right], \tag{S3}$$

where

$$|\Psi_0(\{r_j\})\rangle = M_{r_N}^{(N)} ... M_{r_1}^{(1)} |\psi_0\rangle \otimes |\{r_j\}\rangle, \tag{S4}$$

$$|\Psi_1\rangle = e^{i\delta} |\psi_0\rangle \otimes |\{r_j = -\}\rangle. \tag{S5}$$

Here we introduced the collective state of all the detectors, $|\{r_j\}\rangle$. In the arm denoted by $|1\rangle$ the detectors do not interact with the photon and thus stay in the no-click position.

The second beam splitter converts $|0\rangle \to \frac{1}{\sqrt{2}}(|0\rangle + |1\rangle)$, $|1\rangle \to \frac{1}{\sqrt{2}}(|0\rangle - |1\rangle)$, so that the output state is

$$|\psi\rangle_{out} = \frac{1}{2} \left[\left(|\Psi_1\rangle + \sum_{\{r_j\}} |\Psi_0(\{r_j\})\rangle \right) \otimes |0\rangle + \left(-|\Psi_1\rangle + \sum_{\{r_j\}} |\Psi_0(\{r_j\})\rangle \right) \otimes |1\rangle \right]. \tag{S6}$$

Therefore, the probability of the photon appearing at output ports $|0\rangle$ or $|1\rangle$ is given by

$$\begin{aligned} P_{0/1} &= \frac{1}{2} \left[1 \pm \sum_{\{r_j\}} \mathrm{Re}\, e^{-i\delta} \langle \Psi_1 | \Psi_0(\{r_j\}) \rangle \right] \\ &= \frac{1}{2} \left[1 \pm \mathrm{Re}\, e^{-i\delta} \langle \psi_0 | M_-^{(N)} ... M_-^{(1)} |\psi_0\rangle \right]. \end{aligned} \tag{S7}$$

Note that only the term with no-click readouts, $\{r_j = -\}$, in the measured interferometer arm contributes to the interference, which thus enables the observation of the measurement-induced phase, cf. Eq. (S2) and Section 1 B. In other words, the interference implicitly performs the postselection to $\{r_j = -\}$ required by the definition of $\chi(\theta)$, cf. Section 1 C.

Section 2. EFFECT OF IMPERFECT BIREFRINGENT CRYSTALS

In this section, we discuss some details about the modeling and exploration of the possible defects that may explain the mismatch between the theory and experimental results. While a plethora of plausible defects may occur in our system, it has been assumed that the optical properties of any element correspond to the nominal ones given by the manufacturing companies. Therefore, the study focuses on the effects of minor misalignment on the optical elements, such as incorrect rotation of the QWP at each stage, the imperfect placing of the compensation plate, and non-parallel output beams exiting the Beam displacer. While our preliminary calculations show that these imperfections lead to translation on the transition point. We found that the main contribution to the shift of the transition point is given by effects in which the field's extraordinary and ordinary components exit from the birefringent crystal with slightly non-parallel propagation directions. While the non-parallelism can result from minor misalignment, the beam displacer (THORLABS3 BDY12) manufacturer mentions that both components are parallel to each other within 30 arcseconds. Figure S1-b shows the intensity profile from a YBO_4 crystal when illuminated with diagonally polarized light, and a polariser is placed on the output. The presence of an interference pattern corroborates the existence of a slight deviation in the propagation direction of one of the output beams.

Following these results, we modified the model for our beam displacer to account for our crystals' imperfections. For the sake of a simpler model, higher order optical aberrations have not been considered, despite the intensity profile in Figure S1-b depicts an indication of their presence. First, we assumed that only the extraordinary component experiences a deflection from the optical axis by an angle β. Due to variability from one crystal to another, we allow the deviation to occur along any direction in the transverse plane, characterized by an angle ν. Therefore, it is possible to write the effect of an imperfect beam displacer on an impinging electric field as

$$\mathbf{E}(x,y) = \mathrm{BD}_{\nu,\beta}\, \mathbf{E}_0(x,y) = \sqrt{\frac{2}{\pi w_0^2}} \begin{pmatrix} e^{ikn_y L_y} E_{0y} e^{-(x^2+y^2)/w_0^2} \\ e^{ikn_x L_x + i\Delta(x,y)} E_{0x} e^{-([x-d_x]^2+y^2)/w_0^2} \end{pmatrix} e^{ikz}. \tag{S8}$$

where $\Delta(x,y) = k \sin\beta(\cos\nu x + \sin\nu y)$ implements the deflection on the extraordinary component. Therefore, it is possible to describe the evolution of the input beam $\mathbf{E}_0(x,y)$ due to a sequence of N stages with distinct imperfections as

$$\mathbf{E_u}(x,y) = \prod_{j=1}^{N} \mathcal{M}_{\nu_j,\beta_j}(\theta,\phi_j)\mathbf{E}_0(x,y), \tag{S9}$$

where $\mathcal{M}_{\nu,\beta}(\theta,\phi)$ is obtained by substituting Eq. (S8) into Eq. 9 (Methods Section). A first glance at the effect of these imperfections on the location of the transition is obtained when considering the case when the $N=3$ measurement stages possess identical crystals. As shown in Figure S1c, the location of the topological transition shifts towards higher values of w_0 when the deflection angle increases.

Although the results shown in Figure S1c demonstrate that non-parallel output beams have a dramatic effect on the location of the transition, the assumption of three identical crystals is not valid in our experiment. As a consequence, the behavior of the geometrical phase curve depends directly on three pairs (ν,β), which quantify the non-parallelism between the faces of each BD. A genetic algorithm (GA)[29] was implemented to perform the search for the set of optimal parameters $P = \{\nu_1, \beta_1, \nu_2, \beta_2, \nu_3, \beta_3\}$ that match the experimental results. It should be noted that it is possible to calculate a deviation from the interference pattern in Figure S1b. Additional corrections due to small curvatures in the crystals surfaces could be responsible for residual mismatches between experiment and theory

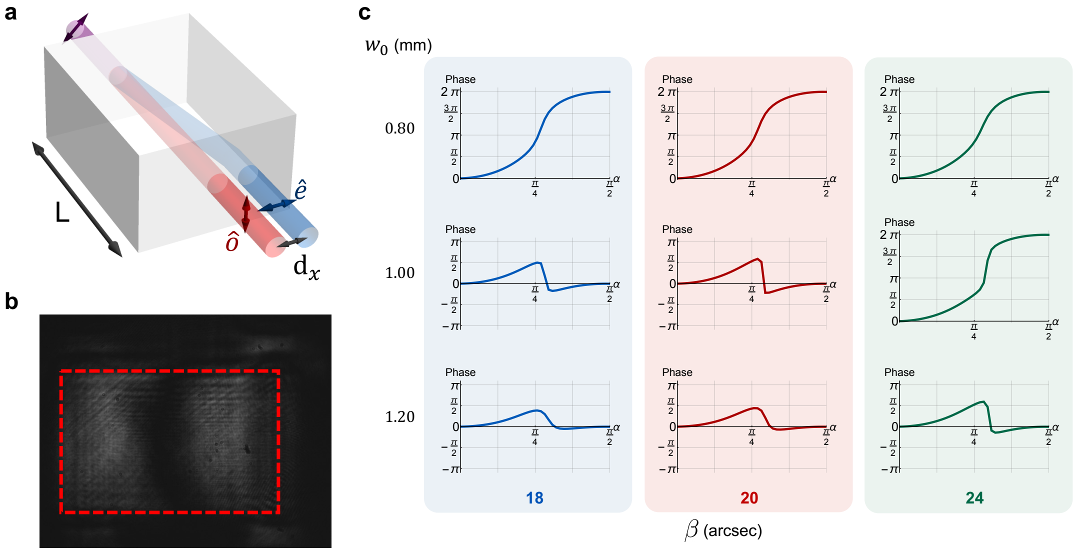

Figure S1. **Modeling an imperfect beam displacer.** **a.** Schematic of a beam displacer (BD). **b.** Interference pattern obtained from the output beams after projecting on circular polarisation (and with a circularly polarized input beam). **c.** Geometrical phase curves for different values of the deviation angle β.

REFERENCES AND NOTES

1. M. V. Berry, Quantal phase factors accompanying adiabatic changes. *Proc. R. Soc. Lond.* **392**, 45–57 (1984).
2. S. Pancharatnam, Generalized theory of interference, and its applications. *Proc. Indian Acad. Sci. A.* **44**, 247–262 (1956).
3. J. Samuel, R. Bhandari, General setting for berry's phase. *Phys. Rev. Lett.* **60**, 2339–2342 (1988).
4. M. V. Berry, S. Klein, Geometric phases from stacks of crystal plates. *J. Mod. Optic.* **43**, 165–180 (1996).
5. D. Chruscinski, A. Jamiolkowski, *Geometric Phases in Classical and Quantum Mechanics* (Birkhäuser Basel, 2004).
6. E. Cohen, H. Larocque, F. Bouchard, F. Nejadsattari, Y. Gefen, E. Karimi, Geometric phase from Aharonov–Bohm to Pancharatnam–Berry and beyond. *Nat. Rev. Phys.* **1**, 437–449 (2019).
7. Z. Bomzon, G. Biener, V. Kleiner, E. Hasman, Space-variant Pancharatnam–Berry phase optical elements with computer-generated subwavelength gratings. *Opt. Lett.* **27**, 1141–1143 (2002).
8. K. Y. Bliokh, M. A. Alonso, M. R. Dennis, Geometric phases in 2d and 3d polarized fields: Geometrical, dynamical, and topological aspects. *Rep. Prog. Phys.* **82**, 122401 (2019).
9. A. Rubano, F. Cardano, B. Piccirillo, L. Marrucci, Q-plate technology: A progress review [Invited]. *J. Opt. Soc. Am. B.* **36**, D70 (2019).
10. C. P. Jisha, S. Nolte, A. Alberucci, Geometric phase in optics: From wavefront manipulation to waveguiding. *Laser & Photonics Reviews* **15**, 2100003 (2021).
11. H. C. Higgins, Öpik, H. C. Longuet-Higgins, U. Öpik, M. H. L. Pryce, R. A. Sack, Studies of the jahn-teller effect. II. the dynamical problem. *Proc. R Soc. Lond.* **244**, 1–16 (1958).
12. J. Zak, Berry's phase for energy bands in solids. *Phys. Rev. Lett.* **62**, 2747–2750 (1989).
13. R. Resta, Manifestations of berry's phase in molecules and condensed matter. *J. Phys. Condens. Matter* **12**, R107–R143 (2000).
14. C. Nayak, S. H. Simon, A. Stern, M. Freedman, S. D. Sarma, Non-abelian anyons and topological quantum computation. *Rev. Mod. Phys.* **80**, 1083–1159 (2008).
15. E. Fradkin, *Field Theories of Condensed Matter Physics* (Cambridge Univ. Press, 2013).
16. R. Resta, Macroscopic polarization in crystalline dielectrics: The geometric phase approach. *Rev. Mod. Phys.* **66**, 899–915 (1994).

17. J. K. Asbóth, L. Oroszlány, A. Pályi, A short course on topological insulators. *Lecture Notes in Physics* **919**, 166 (2016).
18. M. Z. Hasan, C. L. Kane, Colloquium: Topological insulators. *Rev. Mod. Phys.* **82**, 3045–3067 (2010).
19. J. Garrison, E. Wright, Complex geometrical phases for dissipative systems. *Phys. Lett. A.* **128**, 177–181 (1988).
20. G. Dattoli, R. Mignani, A. Torre, Geometrical phase in the cyclic evolution of non-hermitian systems. *J. Phys. A Math. Gen.* **23**, 5795–5806 (1990).
21. R. El-Ganainy, K. G. Makris, M. Khajavikhan, Z. H. Musslimani, S. Rotter, D. N. Christodoulides, Non-Hermitian physics and PT symmetry. *Nat. Phys.* **14**, 11–19 (2018).
22. Y.-W. Cho, Y. Kim, Y.-H. Choi, Y.-S. Kim, S.-W. Han, S.-Y. Lee, S. Moon, Y.-H. Kim, Emergence of the geometric phase from quantum measurement back-action. *Nat. Phys.* **15**, 665–670 (2019).
23. V. Gebhart, K. Snizhko, T. Wellens, A. Buchleitner, A. Romito, Y. Gefen, Topological transition in measurement-induced geometric phases. *Proc. Natl. Acad. Sci. U.S.A.* **117**, 5706–5713 (2020).
24. K. Snizhko, P. Kumar, N. Rao, Y. Gefen, Weak-measurement-induced asymmetric dephasing: Manifestation of intrinsic measurement chirality. *Phys. Rev. Lett.* **127**, 170401 (2021).
25. K. Snizhko, N. Rao, P. Kumar, Y. Gefen, Weak measurement-induced phases and dephasing: Broken symmetry of the geometric phase. *Phys. Rev. Res.* **3**, 043045 (2021).
26. Y. Wang, K. Snizhko, A. Romito, Y. Gefen, K. Murch, Observing a topological transition in weak-measurement-induced geometric phases. *Phys. Rev. Res.* **4**, 023179 (2022).
27. E. Bolduc, N. Bent, E. Santamato, E. Karimi, R. W. Boyd, Exact solution to simultaneous intensity and phase encryption with a single phase-only hologram. *Opt. Lett.* **38**, 3546–3549 (2013).
28. K. Jacobs, *Quantum Measurement Theory and its Applications* (Cambridge Univ. Press, 2014).
29. D. A. Coley, *An Introduction to Genetic Algorithms for Scientists and Engineers* (World Scientific Publishing Company, 1999).
